# Development of SCN Connectivity and the Circadian Control of Arousal: A Diminishing Role for Humoral Factors?

**DOI:** 10.1371/journal.pone.0045338

**Published:** 2012-09-13

**Authors:** Andrew J. Gall, William D. Todd, Mark S. Blumberg

**Affiliations:** Department of Psychology, University of Iowa, Iowa City, Iowa, United States of America; Simon Fraser University, Canada

## Abstract

The suprachiasmatic nucleus (SCN) is part of a wake-promoting circuit comprising the dorsomedial hypothalamus (DMH) and locus coeruleus (LC). Although widely considered a “master clock,” the SCN of adult rats is also sensitive to feedback regarding an animal's behavioral state. Interestingly, in rats at postnatal day (P)2, repeated arousing stimulation does not increase neural activation in the SCN, despite doing so in the LC and DMH. Here we show that, by P8, the SCN is activated by arousing stimulation and that selective destruction of LC terminals with DSP-4 blocks this activational effect. We next show that bidirectional projections among the SCN, DMH, and LC are nearly absent at P2 but present at P8. Despite the relative lack of SCN connectivity with downstream structures at P2, day-night differences in sleep-wake activity are observed, suggesting that the SCN modulates behavior at this age via humoral factors. To test this hypothesis, we lesioned the SCN at P1 and recorded sleep-wake behavior at P2: Day-night differences in sleep and wake were eliminated. We next performed precollicular transections at P2 and P8 that isolate the SCN and DMH from the brainstem and found that day-night differences in sleep-wake behavior were retained at P2 but eliminated at P8. Finally, the SCN or DMH was lesioned at P8: When recorded at P21, rats with either lesion exhibited similarly fragmented wake bouts and no evidence of circadian modulation of wakefulness. These results suggest an age-related decline in the SCN's humoral influence on sleep-wake behavior that coincides with the emergence of bidirectional connectivity among the SCN, DMH, and LC.

## Introduction

The suprachiasmatic nucleus (SCN), a hypothalamic structure critical for the circadian control of arousal, modulates the activity of many downstream structures, including the dorsomedial hypothalamus (DMH) and locus coeruleus (LC) [1]. Although SCN rhythms of metabolic activity are first detectible prenatally at embryonic day (E)19 [2], little is known about the contributions of the SCN and its downstream targets to infant behavior. It is known, however, that day-night differences in sleep and wakefulness are expressed as early as postnatal day (P)2 and that nocturnal wakefulness emerges by P15 [3]. Also by P15, wake bouts, but not sleep bouts, transition from an exponential distribution (for which the probability of a state transition is constant at all bout lengths) to a power-law distribution (which is characterized in part by a small proportion of very long bouts) [3], [4]. Therefore, here we investigate the developmental relations between the SCN-DMH-LC circuit and sleep-wake behavior across the first 3 postnatal weeks, a period of rapid and pronounced changes in sleep-wake organization [5].

The SCN is widely considered a “master clock” that broadcasts circadian signals to the brain and periphery [6], [7]. However, the SCN's spontaneous neural activity is also differentially modulated during sleep and wakefulness [8], thereby indicating feedback control of the SCN. The anatomical pathways through which the SCN receives feedback about vigilance state are not known. One possibility, tested here, is that this feedback is mediated in part by the LC and DMH.

If the LC and DMH do modulate SCN activity, a recent study suggests that they do so only after P2, at least with regard to arousing stimulation. Todd et al. [9] enforced arousal in an infant rat by applying a chilled metal spatula to a region of the snout that contains a high density of cold thermoreceptors [10]. The effect of 30 min of arousing stimulation on neural activity was assessed using Fos immunoreactivity (Fos-ir). Although the LC and DMH were significantly activated by this procedure, the SCN was not. In light of the Deboer et al. [8] findings of state-dependent modulation of SCN activity in adults, as well as evidence that electrical stimulation of the LC in adults directly or indirectly activates the SCN [11], we hypothesized that arousing stimulation would evoke SCN activity sometime after P2.

To test this hypothesis, we repeated the Todd et al. [9] experiment at P8. We found that the SCN, in addition to the DMH and LC, is significantly activated by 30 min of arousing stimulation and that this SCN activation requires a functionally intact LC. Next, we found that bidirectional neural projections among the SCN, DMH, and LC emerge between P2 and P8. Finally, we used lesions and transections to assess the contributions of the SCN and DMH to the consolidation and circadian rhythmicity of sleep and wake bouts before and after the emergence of bidirectional connectivity. Our findings suggest a model of SCN function that includes humoral influences on behavioral state early in development and a predominating influence of direct neural connections by the end of the first postnatal week.

**Figure 1:**
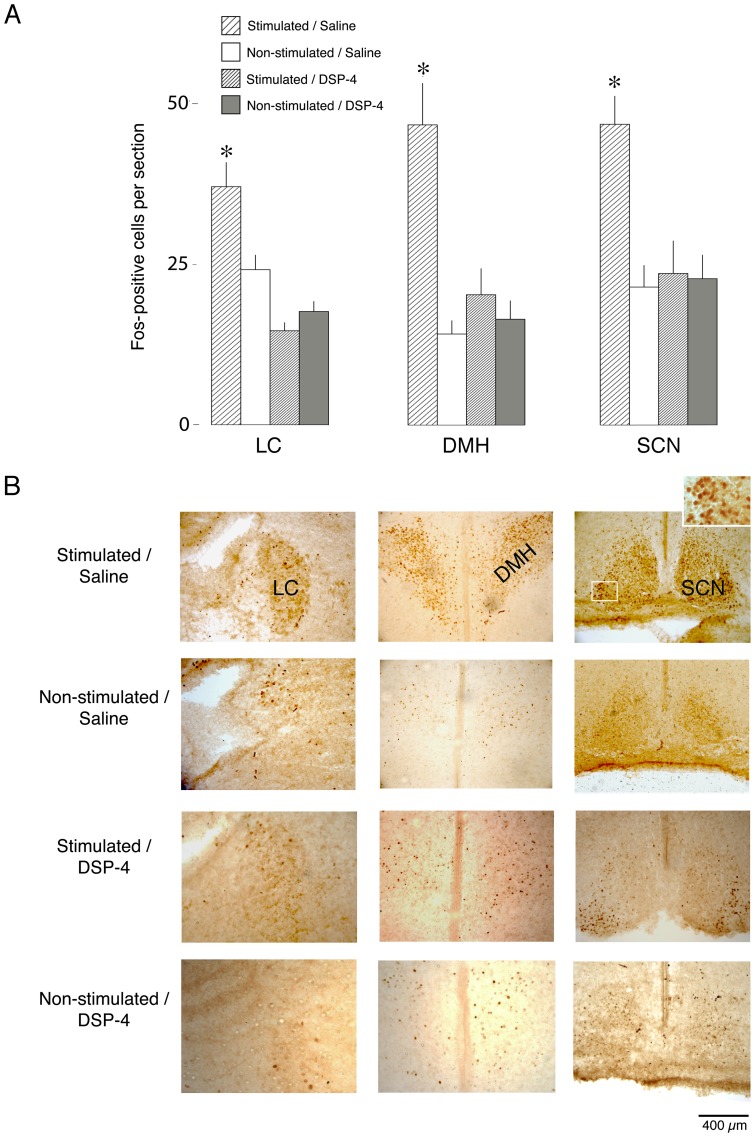
The effect of arousing stimulation at P8 on the SCN-DMH-LC wake-promoting circuit. (A) Mean number of Fos-positive cells per section in the LC, DMH, and SCN in P8 rats of Stimulated/Saline (*n* = 6), Non-Stimulated/Saline (*n* = 6), Stimulated/DSP-4 (*n* = 6), and Non-Stimulated/DSP-4 (*n* = 6) pups. * Significant difference from all other groups. Means + SE. (B) Photographs of the LC, DMH, and SCN in Stimulated/Saline (first row), Non-Stimulated/Saline (second row), Stimulated/DSP-4 (third row), and Non-Stimulated/DSP-4 (fourth row) pups at P8. The inset represents a 100X magnification of cFos+ neurons within the SCN (white square). Abbreviations: LC: locus coeruleus; DMH: dorsomedial hypothalamus; SCN: suprachiasmatic nucleus.

## Materials and Methods

All experiments were carried out in accordance with the National Institutes of Health Guide for the Care and Use of Laboratory Animals (NIH Publication No. 80–23) and were approved by the Institutional Animal Care and Use Committee of the University of Iowa.

**Table 1:** Mean (SE) number of Fos-positive cells per section for the four experimental groups tested at P8.

	Stimulated/Saline	Non-Stimulated/Saline	Stimulated/DSP-4	Non-Stimulated/DSP-4
MPOA	**14.6 (1.5)**	7.9 (1.3)	8.7 (2.6)	8.0 (1.0)
PeF	**17.3 (2.2)**	8.7 (1.9)	9.5 (1.1)	9.7 (1.0)
TMN	**15.0 (1.3)**	8.3 (0.5)	8.0 (1.0)	5.2 (0.6)
Barrel Cortex	**28.5 (5.8)**	8.2 (1.5)	**19.1 (3.4)**	6.8 (1.4)
BF	**27.1 (4.2)**	13.6 (2.0)	**23.0 (4.9)**	12.1 (2.0)
LDT	**24.5 (2.8)**	9.2 (1.0)	**20.8 (2.7)**	8.7 (1.0)
VLPO	6.9 (0.6)	6.9 (0.5)	4.8 (0.4)	6.6 (1.4)
vSPVZ	13.2 (1.3)	9.7 (1.2)	9.4 (0.7)	9.8 (1.2)
MnPO	18.2 (4.0)	13.1 (2.3)	12.2 (2.2)	10.7 (2.5)
LH	14.6 (1.8)	9.3 (1.9)	12.8 (1.6)	10.0 (2.2)
PVN	40.7 (4.7)	37.1 (6.4)	36.8 (5.1)	42.8 (4.5)
DR	13.0 (2.0)	9.8 (2.8)	8.6 (1.7)	7.8 (0.6)
MR	19.7 (3.0)	14.3 (1.8)	14.7 (2.3)	12.4 (1.6)
DTg	24.6 (3.3)	31.0 (2.1)	23.5 (3.2)	27.8 (2.5)
VTg	24.1 (3.4)	15.9 (3.2)	20.7 (2.7)	17.0 (2.5)
PO	11.1 (2.5)	10.3 (1.6)	10.2 (1.1)	10.8 (1.2)

Data for SCN, DMH, and LC are presented in Figure 1.

Bolded values significantly different from unbolded values within each brain area.

Abbreviations: LC: locus coeruleus, DMH: dorsomedial hypothalamus, SCN: suprachiasmatic nucleus, LDT: laterodorsal tegmental nucleus, BF: basal forebrain, PeF: perifornical nucleus, TMN: tuberomammillary nucleus, VTg: ventral tegmental nucleus, MPOA: medial preoptic area, vSPVZ: ventral subparaventricular zone, LH: lateral hypothalamus, VLPO: ventrolateral preoptic nucleus, MnPO: median preoptic nucleus, PO: pontis oralis, DR: dorsal raphe, MR: median raphe, DTg: dorsal tegmental nucleus.

### Subjects

A total of 197 Sprague-Dawley Norway rats (*Rattus norvegicus)* from 109 litters were used in this study. All efforts were made to minimize the number of animals used. For all experiments, males and females were equally represented and littermates were always assigned to different experimental groups. Litters were culled to 8 pups within 3 days of birth (day of birth  = P0). Mothers and their litters were housed and raised in standard laboratory cages (48×20×26 cm) in the animal colony at the University of Iowa. Food and water were available ad libitum. All animals were maintained on a 12-h light-dark schedule with lights on at 0700 h.

**Figure 2:**
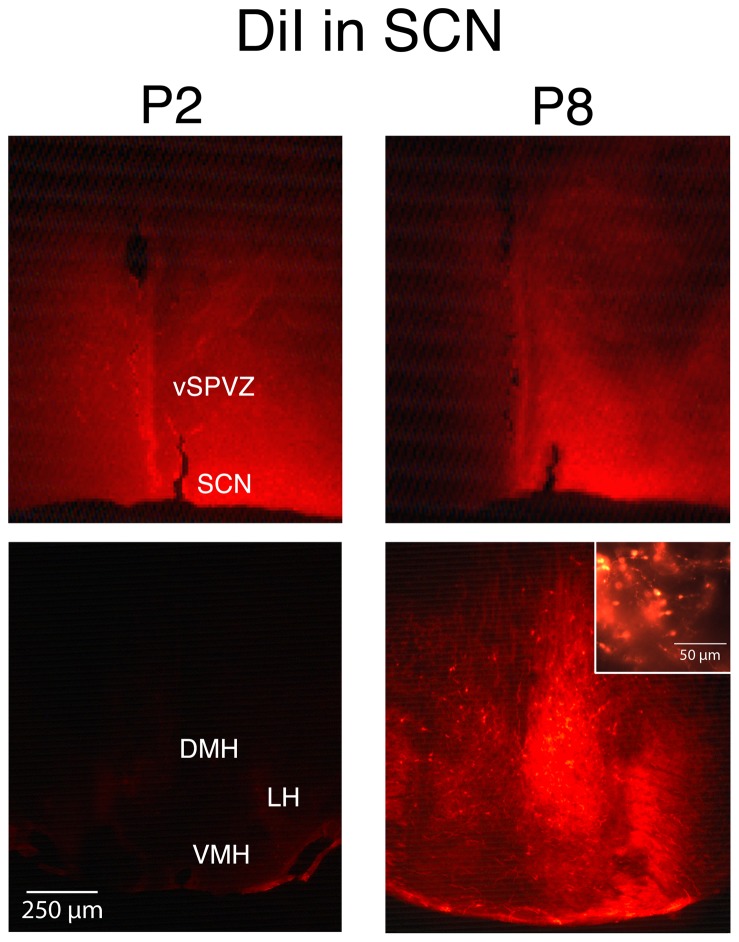
Neural tracing with DiI at P2 and P8 in the SCN. One DiI crystal was placed in the SCN of fixed tissue at P2 (left) and P8 (right). Top row depicts placement of crystal. Bottom row depicts fluorescence in DMH, LH, and VMH. The inset is a magnified view to reveal cell bodies and axon terminals in the DMH. *n* = 4 subjects per group. Abbreviations: DMH: dorsomedial hypothalamus; LH: lateral hypothalamus; VMH: ventromedial hypothalamus; vSPVZ: ventral subparaventricular zone; SCN: suprachiasmatic nucleus.

### Arousing stimulation and Fos immunoreactivity at P8

#### Subjects

Twenty-four infant rats from 6 litters (*n* = 6 per group x4 groups) were used. Littermates were assigned to different experimental groups. At P3 (body weights: 7.2–10.1 g), 2 same-sex pups with visible milk bands were removed from the litter and injected with N-(2-chloroethyl)-N-ethyl-2-bromobenzylamine (DSP-4; 50 mg/kg); the other 2 same-sex pups were injected with saline. DSP-4 is a neurotoxin that selectively destroys noradrenergic LC terminals in infant and adult rats [12]–[14]. Pups were placed back in the litter until recording at P8 (body weights: 16.7–20.1 g; there were no significant group differences in body weight).

**Figure 3:**
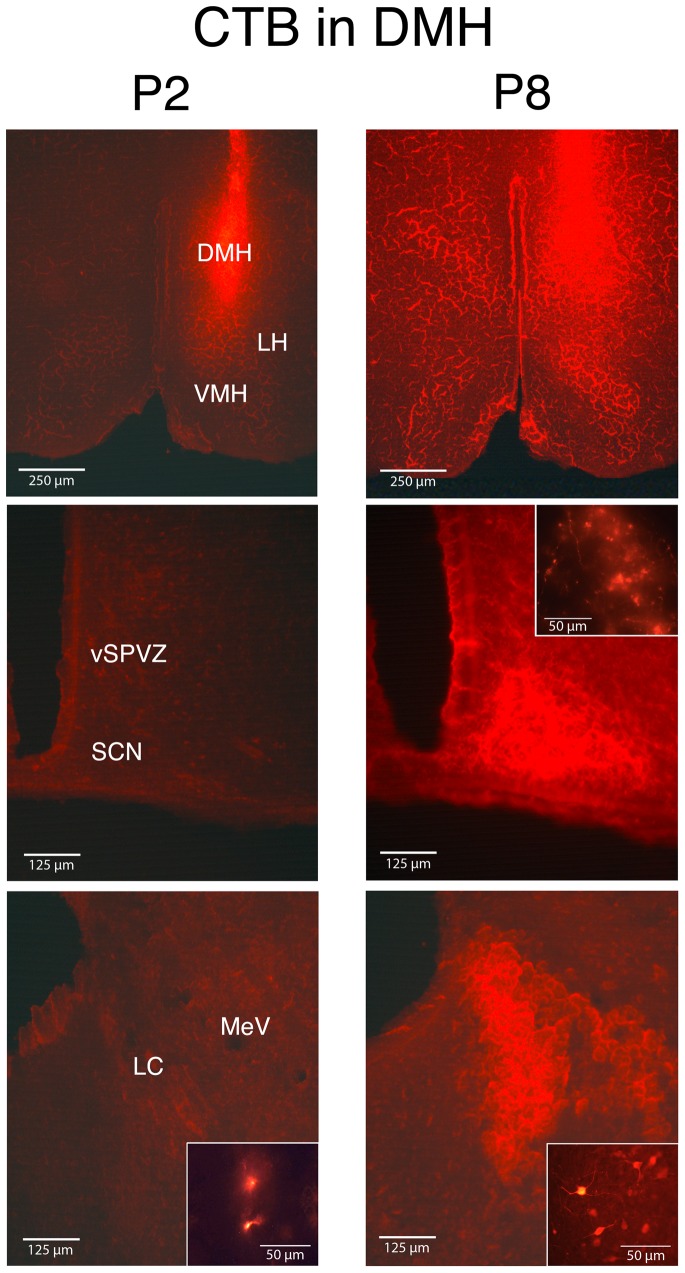
Neural tracing with CTB at P2 and P8 in the DMH. Fluorescent CTB was injected unilaterally (0.1 μL) into the DMH at P2 (left) and P8 (right). Top row depicts the injection site of CTB. Bottom 2 rows depict fluorescence in vSPVZ, SCN, LC, and MeV. The insets are magnified views to reveal cell bodies and axon terminals in the SCN (middle row) and LC (bottom row). *n* = 4 subjects per group. Abbreviations are identical with those of Figure 2, but with the addition of Mesencephalic of V (MeV) and locus coeruleus (LC).

#### Apparatus

The recording chamber consisted of an electrically shielded double-walled glass chamber (height  = 17 cm, i.d.  = 12.5 cm) sealed with a lid. An access hole in the side of the chamber allowed for the passage of electromyographic (EMG) electrodes. Heated water circulated through the walls of the glass chamber to maintain air temperature at thermoneutrality (i.e., 35°C).

**Figure 4:**
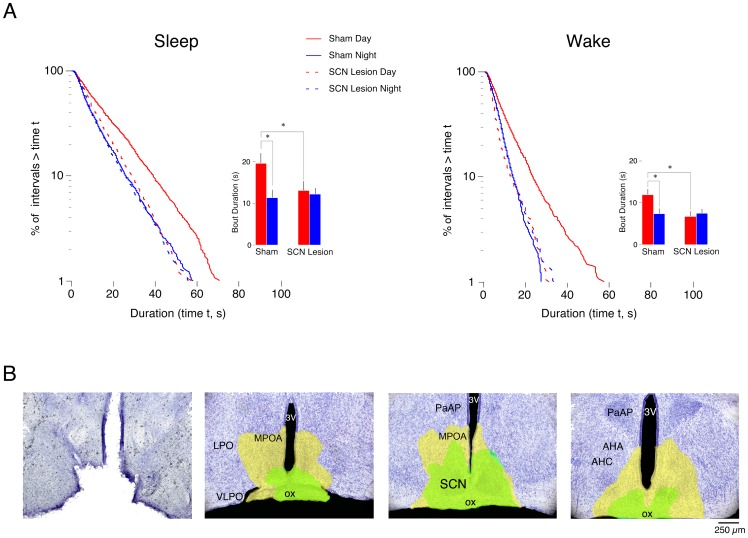
Effects of SCN lesions at P2 on sleep and wakefulness during the day and night. (A) Log-survivor plots of pooled sleep (left column) and pooled wake (right column) bout durations for subjects that experienced sham surgery or SCN lesions at P1 and recorded at P2 (1169–1932 points per plot). Pups were recorded during the day (red) and at night (blue) in shams (solid line) and SCN lesioned pups (dashed line). Insets provide mean sleep and wake bout durations for sham and lesioned pups during the day and at night. *Significantly different. *n* = 5 subjects per group. Means + SE. (B) Photograph of a representative bilateral electrolytic SCN lesion performed at P1 with recording occurring at P2 (left) followed by 3 sequential images (rostral to caudal) depicting the smallest (green filled area) and largest (yellow filled area) lesions per coronal section across all lesioned subjects. Sections are 500 μm apart. Abbreviations: SCN: suprachiasmatic nucleus; 3V: third ventricle; MPOA: medial preoptic area; LPO: lateral preoptic area; VLPO: ventrolateral preoptic area; ox: optic chiasm; PaAP: Anterior part of parvicellular nucleus; AHA: anterior hypothalamic area, anterior; AHC: anterior hypothalamic area, central.

#### Procedure

On the day of recording and under isoflurane anesthesia, 4 littermates were implanted with EMG electrodes in the nuchal muscle. As described previously [15], two bipolar stainless steel electrodes (50 μm diameter, California Fine Wire, Grover Beach, CA) were inserted bilaterally into the nuchal muscles and secured with flexible collodion, Each pup was assigned to one of four experimental groups: Stimulated/Saline, Non-stimulated/Saline, Stimulated/DSP-4, and Non-stimulated/DSP-4. Each pup was lightly restrained in a supine position, transferred to the recording chamber, and allowed 1 h to recover and acclimate.

**Figure 5:**
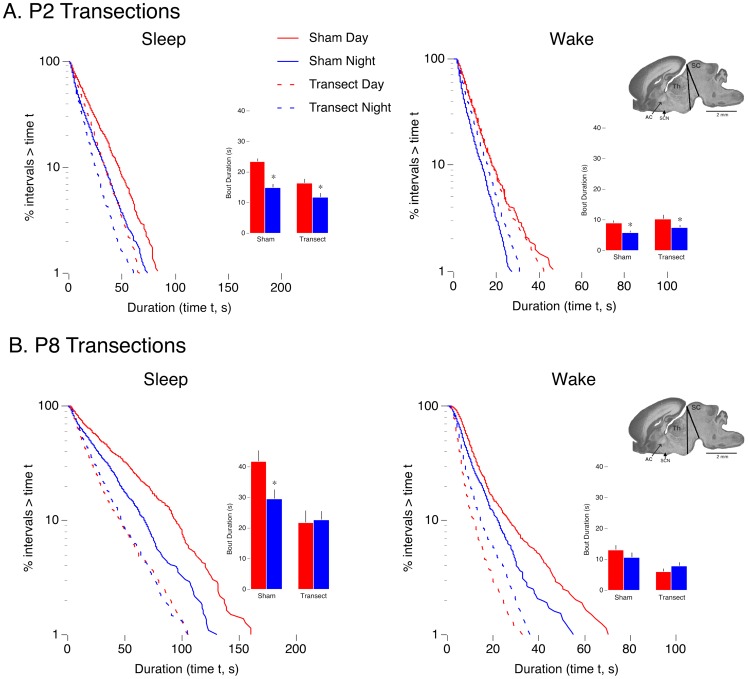
Effects of precollicular transections on day-night differences in sleep and wakefulness. Log-survivor plots of pooled sleep (left column) and pooled wake (right column) bout durations for (A) P2 and (B) P8 subjects that experienced sham surgery or precollicular transections (996–1732 points per plot for P2; 657–1215 points per plot for P8). Sham (solid lines) and transected (dashed lines) pups were recorded during the day (red lines) and night (blue lines). Insets present mean sleep and wake bout durations for sham and lesioned pups during the day and at night. * Significantly different from corresponding daytime value. *n* = 6 subjects per group. Means + SE. The ranges of transections in the sagittal plane are presented at the far right. Abbreviations: AC: anterior commissure; SCN: suprachiasmatic nucleus; Th: thalamus; SC: superior colliculus.

EMG electrodes were connected to differential amplifiers (A–M systems, Carlsborg, WA) and their signals were amplified (x10 k) and filtered (300–5000 Hz). EMG data were acquired (1000 samples/s) and stored using a data acquisition system (BioPac Systems, Santa Barbara, CA). EMG recordings always occurred during the middle of the light period at 1300 h. Each recording consisted of 2 consecutive 30-min periods: a baseline period and a stimulation period. During the baseline period, all pups were allowed to cycle between sleep and wakefulness while EMG data were recorded. During the stimulation period, a cold, metal spatula was applied to the snout [9]. The stimulus was only applied when the subject was asleep, as indicated by nuchal atonia accompanied by behavioral quiescence or myoclonic twitching. Between stimulus presentations, spatulas were kept in a beaker containing ice water. Each stimulus application was recorded in synchrony with EMG data acquisition. Same-sex littermate controls were prepared identically except they were allowed to cycle undisturbed between sleep and wakefulness throughout the 2 30-min periods. As described in our previous study [9], infant animals cycle so quickly between states of sleep and wakefulness that it is not possible to employ a yoked control procedure.

**Figure 6:**
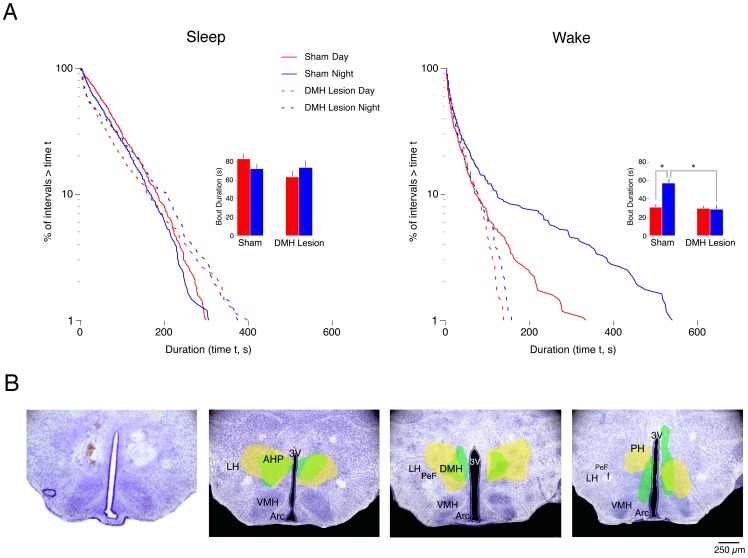
Effects of DMH lesions at P21 on sleep and wakefulness during the day and night. (A) Log-survivor plots of pooled sleep (left column) and pooled wake (right column) bout durations for subjects that experienced sham surgery or DMH lesions at P8 and recorded at P21 (658–921 points per plot). Pups were recorded during the day (red) and at night (blue) in shams (solid line) and DMH lesioned pups (dashed line). Insets provide mean sleep bout durations for sham and lesioned pups during the day and at night. * Significantly different. *n* = 6 subjects per group. Means + SE. (B) Photograph of a representative bilateral ibotenic acid lesion performed at P8 with recording occurring at P21 (left) followed by 3 sequential images (rostral to caudal) depicting the smallest (green filled area) and largest (yellow filled area) lesions per coronal section across all lesioned subjects. Sections are 500 μm apart. Abbreviations: DMH: dorsomedial hypothalamus; 3V: third ventricle; LH: lateral hypothalamus; AHP: anterior hypothalamic area, posterior; VMH: ventromedial hypothalamus; Arc: arcuate nucleus; PeF: perifornical nucleus; PH: posterior hypothalamus; f: fornix.

After the stimulation period, pups were left undisturbed in the chamber for 90 min. They were then removed from the chamber, killed with an overdose of Nembutal, and perfused transcardially with phosphate buffered saline (PBS) followed by 4% paraformaldehyde (PFA). Brains were removed and post-fixed overnight in 4% PFA before being transferred to 30% sucrose solution.

**Figure 7:**
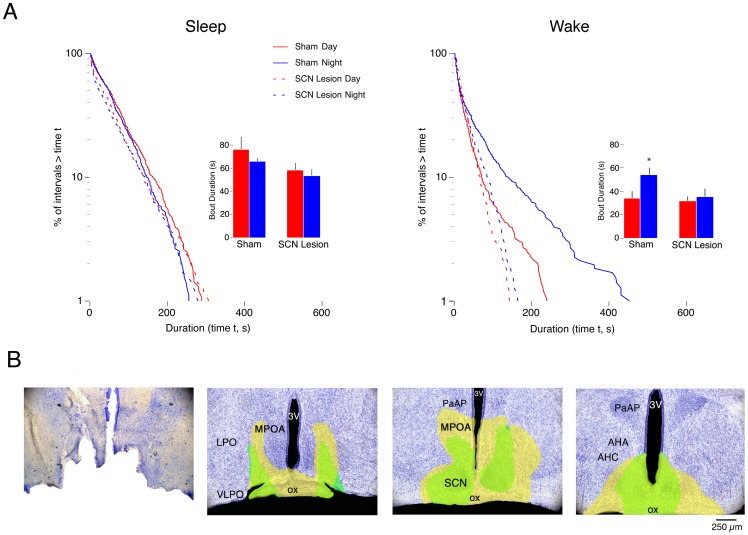
Effects of SCN lesions at P21 on sleep and wakefulness during the day and night. (A) Log-survivor plots of pooled sleep (left column) and pooled wake (right column) bout durations for subjects that experienced sham surgery or SCN lesions at P8 and recorded at P21 (658–937 points per plot). Pups were recorded during the day (red) and at night (blue) in shams (solid line) and SCN lesioned pups (dashed line). Insets provide mean sleep bout durations for sham and lesioned pups during the day and at night. *Significantly different from the corresponding daytime value. *n* = 6 subjects per group. Means + SE. (B) Photograph of a representative bilateral electrolytic SCN lesion performed at P8 with recording occurring at P21 (left) followed by 3 sequential images (rostral to caudal) depicting the smallest (green filled area) and largest (yellow filled area) lesions per coronal section across all lesioned subjects. Sections are 500 μm apart. Abbreviations are identical to Figure 4.

#### Fos immunoreactivity

Coronal sections (40 µm) were cut using a microtome (Model SM 2000 R, Leica, Bensheim, Germany) and placed in wells filled with PBS. Sections were then placed in normal goat serum for 1 h, rinsed again with PBS, and incubated in a primary antibody solution (1∶2000, sc-7202, in .01 M PBS and 0.3% Triton X; Santa Cruz Biotechnology, Santa Cruz, CA). After 24 h, sections were rinsed with PBS and incubated in biotinylated goat anti-rabbit IgG secondary antibody (1∶200; Vector Laboratories, Burlingame, CA) for 1 h in .01 M PBS and .3% Triton X. Sections were rinsed with PBS and placed in an avidin-biotin peroxidase complex (Vector Laboratories) for 1 h. The sections were again rinsed with PBS before being placed in a diaminobenzidine solution with hydrogen peroxide (Sigma, St. Louis, MO). The sections were placed in PBS to stop the reaction. Sections were mounted and coverslipped.

**Figure 8:**
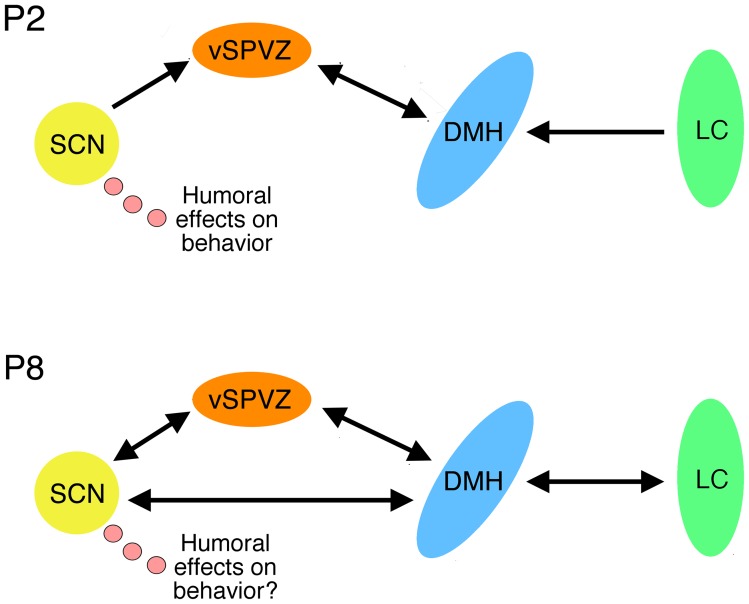
Model of SCN-DMH-LC developing circuitry between P2 and P8. Neural projections among the SCN, vSPVZ, DMH, and LC at P2 are sparse, whereas bidirectional projections among all areas develop by P8. The development of neural connectivity between the SCN and downstream structures may be associated with decreased contributions of SCN-produced humoral factors on sleep-wake behavior.

#### Data Analysis

The number of stimulus presentations was quantified for all 6 5-min segments during the 30-min stimulation period. A microscope (Leica Instruments, Wetzlar, Germany) and imaging system were used to visualize 40 μm brain sections using a 20x objective. Images were imported into ImageJ (National Institutes of Health) and adjusted to binary values.

Using methods similar to those used previously [9], for each subject Fos-positive cells were counted unilaterally in 2–3 sections using a counting box of known dimensions. The experimenter was blind to experimental group. An Abercrombie correction was performed for each area within each section [16]. The mean number of Fos-positive cells per brain area was then calculated for each pup.

In addition to the LC, DMH, and SCN, other areas were selected for quantification. The barrel cortex was selected because it receives somatosensory inputs from the whisker pad [17]; therefore, this area was used as a positive control to demonstrate the sensitivity of the Fos-ir method for detecting somatosensory stimulation of the snout. The following additional wake-related brain areas and nuclei [18]–[23] were selected for quantification: laterodorsal tegmental nucleus (LDT), lateral hypothalamus (LH), basal forebrain (BF), ventral tegmental nucleus (VTg), dorsal tegmental nucleus (DTg), medial preoptic area (MPOA), perifornical area (PeF), and tuberomammillary nucleus (TMN). The following sleep-related brain areas and nuclei [20], [24], [25] were also selected: nucleus pontis oralis (PO), median preoptic nucleus (MnPO), ventrolateral preoptic nucleus (VLPO), dorsal raphe (DR), and median raphe (MR). Finally, the ventral subparaventricular zone (vSPVZ), which is connected to the SCN and DMH [19] and is important for circadian rhythms of sleep and wakefulness [26], was also selected, as was the paraventricular nucleus (PVN), a hypothalamic nucleus important for stress responses [27].

A two-way analysis of variance (ANOVA) was used to analyze group differences for each area sampled. Fisher's PLSD was used as a post hoc test. Alpha was set conservatively at .01 to compensate for multiple comparisons.

### DiI and CTB tracing

#### DiI crystal in fixed tissue

Four P2 (body weights: 6.9–9.2 g) and 4 P8 (body weights: 17.1–19.9 g) rats from 8 litters were used. Pups were removed from the litter during the middle of the lights-on period (1430 h), killed, and perfused as described above. Brains were extracted and allowed to soak in 4% PFA for at least 24 h. Each brain was then removed from the PFA solution and placed on its dorsal surface under a dissecting microscope. Using forceps, a FAST DiI crystal (Molecular Probes, Eugene, OR) was carefully inserted unilaterally within the SCN, which is immediately dorsal to the optic chiasm. Placement of the crystal was counterbalanced such that 4 subjects had a crystal placed into the left side of the SCN, and 4 subjects had a crystal placed into the right side of the SCN. Each brain was then placed back in the PFA solution and allowed to soak for 6 weeks in a dark glass jar at 4°C [28], after which remaining crystal was removed with a forceps. Using a freezing microtome, brains were sliced in 40 µm sections and coverslipped using Vectastain hard set (Vector Laboratories, Burlingame, CA). Every other slice was used for fluorescence analysis. Remaining sections were stained with cresyl violet.

#### CTB in vivo

Four P2 (body weights: 7.0–8.8 g) and 4 P8 (body weights: 16.0–20.4 g) rats from 8 litters were used. Under isoflurane anesthesia, pups were secured in a stereotaxic instrument. A small hole was drilled at the following coordinates: AP: −2.2 mm caudal to bregma, ML: 0.2 mm from midline, DV: −6.0 mm from the surface of the brain. Placement of the syringe was counterbalanced between the left and right DMH. A 30 gauge needle attached to a 1 µl Hamilton syringe was filled with Alexa Fluor 594 (Texas-Red)-conjugated Cholera Toxin B Subunit (CTB) (1 mg/ml; Molecular Probes, Eugene, OR, USA). Once the target area was reached, the needle was left in place for 1 min, after which 0.1 µl of CTB was infused over 30 s. After the injection, the needle remained in the brain for 1 min and then removed slowly. Vetbond was used to close the incision and peanut oil was brushed lightly on the scalp to deter the mother from reopening the wound. The pup recovered for at least 2 h in a humidified incubator maintained at thermoneutrality and then placed back in the litter. Three days later, pups were removed from the litter during the middle of the lights-on period (1430 h), killed, and perfused as described above.

#### Data Analysis

Sections were analyzed for the presence or absence of DiI or CTB labeling (i.e., fluorescent cell bodies and/or axon terminals) using a fluorescent microscope and imaging system (Leica Instruments, Wetzlar, Germany). Fluorescent sections were directly compared to adjacent counterstained Nissl sections. Images of fluorescent and Nissl sections were overlayed using Adobe Photoshop to determine the location of DiI or CTB labeling.

For each subject, fluorescence was quantified unilaterally in 1–4 sections per brain area. Fluorescent images were imported into ImageJ (National Institutes of Health) and the mean red intensity was analyzed using the RGB Measure plugin. For subjects in which a DiI crystal was implanted in the SCN, mean fluorescence intensity was analyzed within the DMH and LC. For subjects in which CTB was injected into the DMH, mean fluorescence intensity was analyzed within the SCN and LC. The experimenter was blind to experimental group and age. Unpaired t tests were used to analyze age group differences (i.e., P2 vs. P8) in mean fluorescence intensity. Alpha was set at .05.

### SCN lesions at P1

#### Procedure

Twenty rats from 10 litters (n = 5 per group with 2 recording times x 2 conditions) were used. Littermates were assigned to different experimental groups. On P1 (body weights: 5.9–8.4 g), a subject with a visible milk band was removed from the litter and, beginning at 1000 h, anesthetized with isoflurane and secured in a stereotaxic apparatus (David Kopf Instruments, Tujunga, CA, USA). Using an insulated tungsten concentric bipolar electrode (1 MΩ, 3–4 μm at the tip, Model TM33CCINS, World Precision Instruments, Sarasota, FL, USA), bilateral electrolytic lesions were made in the SCN. Electrolytic lesions were necessary because SCN neurons are resistant to excitotoxicity 29]. The following coordinates were used: AP: −0.2 mm from bregma, ML: ±S0.1 mm, and DV: −4.5 mm ventral to the meningeal surface. Lesions were made using a stimulus generator (Model SD9F, Grass Technologies, Quincy, MA, USA) and a linear stimulus isolator (Model A395R, World Precision Instruments, Sarasota, FL, USA) to deliver 1.0 mA of DC current for 30 s. Vetbond was used to close the incision and peanut oil was brushed lightly on the scalp. The sham control group experienced the same procedure except that current was not applied. EMG electrodes were implanted as described above.

After surgery, pups recovered for at least 2 h inside a humidified incubator maintained at 34–35°C. Subjects were then placed back in the litter. On the next day at P2 (body weights: 6.8–9.4 g; there were no significant group differences in body weight), subjects were recorded during the day (1200–1400 h) or night (2400–0200 h), with order of recording time counterbalanced. Pups were recorded for sleep and wakefulness using methods identical to those described above. At the conclusion of recording, pups were overdosed with sodium pentobarbital and perfused transcardially, as described above.

#### Histology

Brains were sliced in 50 μm sections using a freezing microtome. Brain slices were stained with cresyl violet. The extent of the lesion was determined by examining sequential brain slices.

#### Data Analysis

EMG data were analyzed using AcqKnowledge software (BioPac Systems, Santa Barbara, CA). EMG signals were integrated and full-wave rectified, and dichotomized into high muscle tone and atonia (or wake and sleep, respectively), as described previously [30]. Data were scored by an individual blind to experimental condition. Sleep and wake bout durations were imported into Statview 5.0 (SAS Institute, Cary, NC) for analysis. Mean sleep and wake bout durations were determined for each subject [3], [31]. Percentage of time awake was calculated by dividing the mean wake bout duration by the sum of the mean sleep and wake bout durations, and then multiplying by 100.

As described previously [4], log-survivor distributions of sleep and wake bout durations were produced from individual and pooled data. To test whether pooled bout duration data were better fit by a power-law or exponential model, log-likelihood functions were calculated and maximized for each model [32]–[34]. The comparison was quantified by calculating Akaike weights, w*_i_* (*i* = 1,2), for each model: w*_i_* = exp(−Δ*_i_*/2)/(exp(−Δ_1_/2) + exp(−Δ_2_/2)). Δ*_i_* is a measure of each model relative to the better model; Δ*_i_*  = 0 for the model with larger log-likelihood value. For the other model, Δ*_i_* >0 is calculated as the difference of Akaike Information Criterion (AIC) values, AIC*_i_*  = −2(log-likelihood*_i_*) +2*k_i_*, where *k_i_* is the number of parameters in model *i*. The Akaike weight for a model estimates the probability that the model is the better of the two candidate models [32], [35]. A model with a weight of 1 is a better fit than a model with a weight of 0.

A two-way ANOVA was used to test for differences across groups (i.e., lesion vs. control) and recording times (i.e., day vs. night). If an interaction was significant, post hoc tests using Fisher's PLSD were performed. Planned comparisons (i.e., unpaired t tests) were used to test for day-night differences within groups. Alpha was set at 0.05 and Bonferroni corrections were used when appropriate. Means are presented with standard errors.

### Precollicular transections at P2 and P8

#### Procedure

Twenty-four P2 rats (body weights: 6.9–9.2 g) from 6 litters (n = 6 per group with 2 recording times x 2 conditions) and 24 P8 rats (body weights: 16.8–21.2 g) from 6 litters (n = 6 per group with 2 recording times x 2 conditions) were used. Littermates were assigned to different experimental groups. On the day of recording, a pup with a visible milk band was removed from the litter. Under isoflurane anesthesia, precollicular transections were made by drilling a small hole in the skull approximately 3 mm caudal to lambda, and then manually inserting a blunted 25 g needle to the base of the brain and rotating the needle using a side-to-side motion [30], [31]. Sham littermates of the same age underwent anesthesia and drilling, but the needle was not inserted. Vetbond was used to close the incision and peanut oil was brushed lightly on the scalp. Next, two bipolar stainless steel electrodes (50 μm diameter, California Fine Wire, Grover Beach, CA) were inserted bilaterally into the nuchal muscles and secured with flexible collodion [15]. After surgery, pups recovered in a humidified incubator maintained at thermoneutrality (34–35°C) for at least 1 h before recording.

Subjects were recorded in pairs in 2 identical electrically shielded double-walled glass chambers during the day (1200–1400 h) and at night (2400–0200 h), with order of recording time counterbalanced. Air temperature in the chambers was regulated at thermoneutrality (i.e., 35.5°C). Nuchal electrodes were connected to differential amplifiers (A–M systems, Carlsborg, WA) and their signals were amplified (x10k) and filtered (300–5000 Hz). EMG data were acquired (1000 Hz) and stored using a data acquisition system (BioPac Systems, Santa Barbara, CA).

Immediately after recording, all subjects were overdosed with sodium pentobarbital and perfused transcardially with PBS followed by 3% formalin. Heads were post-fixed in a sucrose-formalin solution for at least 24 h.

#### Histology

The rostrocaudal placement of the transection was determined using gross visual inspection of each brain and drawn onto a photomicrograph of a P2 or P8 sagittal section.

#### Data Analysis

Data analysis was similar to that described above for the SCN lesions at P2.

### SCN and DMH lesions at P8

#### Procedure

Forty-eight rats from 24 litters (n = 6 per group with 2 recording times x 4 conditions) were used. Littermates were assigned to different experimental groups. At P8 (body weights: 17.4–20.5 g), a subject with a visible milk band was removed from the litter. Under isoflurane anesthesia and using a stereotaxic apparatus, bilateral lesions were produced either in the SCN or DMH.

For SCN lesions, the following electrode coordinates were used: AP: −0.2 mm from bregma, ML: ±0.1 mm, and DV: −5.5 mm ventral to the meningeal surface. A littermate control experienced the same procedures except that current was not applied. For DMH lesions, 0.1 µl of a 10% ibotenic solution (Sigma, St. Louis, MO) was injected bilaterally using the following coordinates AP: −2.2 mm caudal to bregma, ML: ±0.2 mm from midline, DV: −6.0 mm from the surface of the brain. The rate of infusion was 0.1 µl / 30 s. A littermate control experienced the same procedure except 0.1 µl of saline was injected bilaterally.

After lesions were produced, Vetbond was used to close the incision and peanut oil was brushed lightly on the scalp. Pups were placed in an incubator for at least 2 h to recover, and then placed back in the litter. On the day of recording at P21 (body weights: 52.4–69.7 g; there were no significant group differences in body weight), one lesioned pup and one control pup was placed in separate humidified chambers maintained at thermoneutrality for pups at this age (i.e., 29°C). Littermate pairs were recorded for 6 h either during the day (1000–1600 h) or at night (2200–0400 h), with order of recording time counterbalanced across pairs. After recording, pups were killed and perfused as described above. Brains were postfixed and sliced to observe the extent of the lesions, as described above.

#### Data Analysis

Data analysis was similar to that described above for the SCN lesions at P2.

Unless otherwise noted, all means are presented with their standard errors.

## Results

### At P8, DSP-4 blocks the activating effects of arousing stimulation on the SCN

As reported previously, arousing stimulation in P2 rats resulted in increased Fos-ir in the LC and DMH, but not the SCN [9]. Here, at P8, Stimulated/Saline pups expressed significantly more Fos-ir in the LC and DMH, and also in the SCN, as compared with all other conditions (Figure 1A). For the LC, DMH, and SCN, ANOVAs revealed significant main effects of group (i.e., saline vs. DSP-4; F_1,20_s >6.8, Ps<.05) and condition (i.e., stimulated vs. non-stimulated; F_1,20_s >4.4, Ps<.05), and significant group x condition interactions (F_1,20_s >8.3, Ps<.01). Therefore, DSP-4 was effective in blocking sensory-related activation of the SCN. Figure 1B depicts representative images of the LC, DMH, and SCN in the 4 experimental conditions.

Table 1 presents the mean number of Fos-positive cells in other areas of interest for all 4 experimental groups at P8. Three patterns of results are seen: First, in addition to LC, DMH, and SCN, there were 3 areas exhibiting significantly higher Fos-ir in the Stimulated/Saline group compared with the three other groups; this pattern reflects sensory processing that is dependent on the LC. Second, there were areas (e.g., barrel cortex) exhibiting significantly higher Fos-ir in the stimulated groups compared with the non-stimulated groups; this pattern reflects sensory processing that is not dependent on the LC. Third, there were areas (e.g., DTg, MnPO, VLPO) that were not affected by any of the manipulations.

It is possible that stress associated with the stimulation procedure contributed to the increased Fos-ir in the SCN, DMH, and LC. We tested this possibility by measuring Fos-ir in the PVN, an important central component of the stress response [27]. We found no evidence of increased Fos-ir in the PVN in the Stimulated/Saline or Stimulated/DSP-4 groups in relation to the Non-Stimulated groups (see Table 1).

Similar to previous results [9], P8 saline-treated subjects required significantly more stimulus presentations over the last 5 min of the stimulation period (46.3±4.6 stimulations) as compared to the first 5 min of the stimulation period (17.8±2.4 stimulations) to keep them awake (*t*_10_  = 9.8, P<.001), a sign of increased sleep pressure. This was not the case, however, for DSP-4-treated pups (22.5±3.5 stimulations in first 5 min as compared to 23.5±3.9 stimulations in last 5 min; *t*_10_  = 0.6, *NS*). Nonetheless, the total number of stimulus presentations over the 30-min stimulation period was not significantly different between saline-treated (161.9±14.8) and DSP-4-treated (149.3±11.2) pups (*t*_10_  = 1.3, *NS*). A repeated-measures ANOVA over the entire 30-min stimulation period revealed a significant effect of time (F_5,60_  = 6.9, P<.0001) and a significant group x time interaction (F_5,60_  = 7.1, P<.0001), but not a significant effect of group (F_1,10_ = 3.9, *NS*).

### Bidirectional projections within the SCN-DMH-LC circuit develop between P2 and P8

Given the evidence presented above of increasing functional connectivity among the SCN, DMH, and LC between P2 and P8, we next determined whether direct neural projections develop among these 3 structures between P2 and P8.

DiI crystal placed unilaterally within the SCN revealed a developmental increase in connectivity with the DMH (Figure 2). This observation was supported by measurement of mean fluorescent intensity, which was significantly higher at P8 than at P2 in the DMH (*t*_6_  = 10.7, P<.0001) but not the LC (*t*_6_  = .55, *NS*). At P8, both cell bodies and axon terminal were evident in the DMH (Figure 2, inset), thus indicating that bidirectional projections develop between the SCN and DMH by this age. In contrast, very little fluorescence within the LC was observed at either age.

Beyond the DMH and LC, at P2 we observed axon terminals within the lateral septum (LS), MPOA, and vSPVZ. At P8, we observed cell bodies within the vSPVZ and axon terminals within the LH, ventromedial hypothalamus (VMH), vSPVZ, LS, MPOA, and supraoptic nucleus (SON).

Unilateral injection of CTB into the DMH revealed a developmental increase in connectivity with the SCN and LC (Figure 3). In support of these observations, fluorescent intensity was significantly higher at P8 than at P2 in the SCN (*t*_6_ = 7.3, P<.0005) and LC (*t*_6_ = 8.5, P<.0001). At P2, cell bodies were evident within the LC, whereas at P8 cell bodies and axon terminals were detected in both the SCN and LC (Figure 3, insets), thus indicating the development of bidirectional projections linking each of these two structures with the DMH.

Beyond the SCN and LC, at P2 we observed cell bodies and axon terminals within the vSPVZ and axon terminals within the paratenial thalamus (PT) and VMH. At P8, we observed axon terminals within the PT, VMH, DR, DTg, VTg, LH, VLPO, and paraventricular nucleus (PVN).

Two pups received injections of CTB that missed the DMH region. In one pup at P2, the injection was in an undefined region dorsal to the DMH and ventral to the thalamic nucleus submedius, resulting in no visible fluorescent labeling anywhere in the brain other than the injection site. In a second DMH “miss” at P8, the CTB injection site was within the medial tuberal nucleus, just lateral and dorsal to the DMH; this resulted in fluorescent axon terminals in the dorsal raphe nucleus, dorsal tegmental nucleus, and motor trigeminal nucleus. Importantly, for both of these misses, fluorescent labeling was very different from that seen with injections that hit the DMH.

### SCN lesions at P1 eliminate day-night differences in sleep and wakefulness

Although the SCN has limited direct or indirect connectivity with the brainstem at P2, day-night differences in sleep and wakefulness are detectible at that age [3]. To determine if the SCN is critical for this early expression of day-night differences in sleep and wakefulness, we performed SCN lesions at P1 and recorded EMG activity in pups at P2.

Log-survivor distributions for pooled sleep and wake bout durations for sham and SCN-lesioned pups are presented in Figure 4A. Straight lines on these semi-log plots are indicative of exponential distributions [4], [36]. Based on Akaike weight analyses of pooled and individual data (see Methods), sleep and wake bout for shams and SCN-lesioned pups distributed exponentially during the day and night (Akaike weights equal to 1).

The insets in Figure 4A present mean sleep and wake bout durations in sham and SCN-lesioned pups. For shams, and consistent with previous findings [3], mean sleep and wake bout durations were shorter at night than during the day. In contrast, SCN-lesioned pups did not exhibit significant day-night differences in mean sleep or wake bout durations. For wake bout durations, ANOVA revealed a significant main effect of recording time (F_1,16_ = 9.0, P<.01) and a significant group x recording time interaction (F_1,16_ = 5.7, P<.05), but not a significant main effect of group (F_1,16_ = 3.5, *NS*). For sleep bout durations, ANOVA revealed a significant main effect of group (F_1,16_ = 6.3, P<.05) and a group x recording time interaction (F_1,16_ = 6.8, P<.05), but not a significant main effect of recording time (F_1,16_ = 3.6, *NS*). In addition, post hoc tests revealed that during the day, mean sleep and wake bout durations differed significantly between sham and SCN-lesioned groups.

Figure 4B shows the extent of the lesions. The smallest (green filled) and largest (yellow filled) lesions across all pups are presented. Adjacent areas to the SCN were also affected by the electrolytic lesions in some pups, including parts of the optic chiasm and MPOA.

Six pups from 6 separate litters received electrolytic lesions that missed the SCN and spared all cells within it. In these cases, lesions were observed within the MPOA and PVN. Importantly, pups with these “misses” exhibited day-night differences in sleep and wakefulness that were similar to those of shams.

A total of 4 SCN-lesioned pups died before recording at P2 (mortality rate  = 13.3%). Finally, no immediately obvious behavioral differences were detected in lesioned pups as compared to controls.

### Precollicular transections at P2 and P8 exert different effects on day-night differences in sleep and wakefulness

Given that the SCN is functional at P2 but lacks connectivity with downstream structures, it could be that its influence on sleep and wakefulness is mediated humorally. To test this possibility, we performed precolllicular transections at P2 and P8 to physically isolate the SCN from the brainstem.

Figure 5 presents log-survivor distributions for pooled sleep and wake bout durations for transected and sham P2 and P8 rats recorded during the day or night. Based on Akaike weight analyses of pooled and individual data, all sleep and wake data distributed exponentially (Akaike weights equal to 1). The range of the transections at each age is also shown. Note that all precollicular transections were caudal to the SCN and DMH.

At P2 (Figure 5A), log-survivor distributions indicate continued expression of day-night differences in sleep and wake bouts after precollicular transection. Mean sleep and wake bout durations are presented as insets in Figure 5A. For mean sleep bouts, ANOVA revealed significant main effects of group (F_1,20_ = 13.6, P<.005) and recording time (F_1,20_ = 22.4, P<.001), but not a significant group x recording time interaction (F_1,20_ = 1.9, *NS*). For wake bouts, ANOVA revealed a significant main effect of recording time (F_1,20_ = 9.5, P<.01), but no significant main effect of group (F_1,20_ = 2.9, *NS*) or group x recording time interaction (F_1,20_ = .04, *NS*).

At P8 (Figure 5B), precollicular transections eliminated day-night differences in sleep bouts. However, consistent with previous observations at P8 [3], wake bouts did not exhibit day-night differences in sham subjects; thus, day-night differences in wake bouts could not be eliminated by the transections. Mean sleep and wake bout durations are presented as insets in Figure 5B. For sleep bouts, ANOVA revealed significant main effects of group (F_1,20_ = 16.3, P<.001), but no significant effects for recording time (F_1,20_ = 2.9, *NS*) and not a significant group x recording time interaction (F_1,20_ = 3.9, *NS*). For wake bouts, ANOVA revealed a significant main effect for group (F_1,20_ = 14.9, P<.005), but no significant effect of recording time (F_1,20_ = 0.06, *NS*) and no significant group x recording time interaction (F_1,20_ = 2.5, *NS*).

Two P2 transected pups died before recording (mortality rate  = 7.7%) and 3 P8 pups died before recording (mortality rate  = 11.1%). No immediately obvious behavioral differences were detected in transected pups as compared to shams.

### DMH lesions at P8 prevent day-night differences in wakefulness at P21

The precollicular transections described above suggest a loss of SCN humoral control of sleep-wake behavior by P8. To explore this possibility using a more precise method, we performed DMH lesions at P8 and recorded EMG activity at P21, an age by which nocturnal wakefulness and power-law wake bout distributions have emerged [3], [4]. Because the DMH is a major neural output of the SCN, elimination of day-night differences would provide further support for the notion that the SCN relies on direct neural connectivity to modulate sleep and wakefulness after P8.

Figure 6A presents log-survivor distributions for pooled sleep and wake bout durations for DMH-lesioned and sham pups. Whereas sleep bout distributions were almost identical in lesioned and sham pups, wake bout distributions in the lesioned pups, in comparison with those of the shams, were highly and similarly fragmented during the day and night. Also, as expected for shams at P21 [3], [4], [36], Akaike weight analyses of pooled and individual data showed that sleep bouts followed an exponential distribution and wake bouts followed a power-law distribution. But for pups with DMH lesions, all distributions followed an exponential distribution. All individual and pooled Akaike weights were equal to 1.

The insets in Figure 6A provide mean sleep and wake bout durations in DMH-lesioned and sham pups. These data support the conclusions based on the log-survivor data. For mean sleep bout durations, ANOVA revealed no significant main effects for group (F_1,20_ = 2.2, *NS*) or recording time (F_1,20_ = 0.002, *NS*), and no significant group x recording time interaction (F_1,20_ = 3.1, *NS*). In contrast, for mean wake bout durations, ANOVA revealed a significant main effect of group (F_1,20_  = 14.5, P<.005) and recording time (F_1,20_ = 10.3, P<.005), and a significant group x recording time interaction (F_1,20_ = 12.1, P<.005). Post hoc tests revealed a significant difference between mean wake bout durations at night in DMH-lesioned pups as compared with shams. Also, consistent with previous findings [3], percentage of time awake at night was significantly increased in shams but not in lesioned pups. ANOVA revealed no main effect of group (F_1,20_  = 3.2, *NS*), but did reveal a significant main effect of recording time (F_1,20_ = 4.4, P<.05) and a significant group x recording time interaction (F_1,20_ = 11.2, P<.005).

Figure 6B shows the extent of the chemical lesions in the DMH. In addition to damage to the DMH, adjacent areas were also affected in some pups, including parts of the anterior hypothalamus (AHP) and posterior hypothalamus (PH).

Six additional pups from 6 litters received lesions that spared all cells within the DMH. In these cases, lesions were found in the anterior hypothalamic area and posterior hypothalamus. These “misses” exhibited day-night differences in sleep and wakefulness and power-law distributions of wakefulness that were similar to controls.

A total of 6 DMH-lesioned pups died before recording at P21 (mortality rate  = 16.7%). Finally, no immediately obvious behavioral differences were detected in lesioned pups as compared to controls.

### SCN lesions at P8 prevent day-night differences in wakefulness at P21

Figure 7 presents the effects of SCN lesions at P8 on day-night difference in sleep and wakefulness in pups recorded at P21. In all substantive ways, SCN lesions produced effects similar to those of the DMH lesions described above. Log-survivor distributions for pooled sleep and wake bout durations for SCN-lesioned pups and shams are presented in Figure 7A. Again, SCN lesions had little effect on sleep bout durations but produced substantial fragmentation of wake bouts and eliminated their power-law distribution.

The insets in Figure 7A present mean sleep and wake bout durations in SCN-lesioned pups and shams. For mean sleep bout durations, ANOVA revealed a significant main effect of group (F_1,20_  = 4.9, P<.05), but not a significant main effect of recording time (F_1,20_  = 1.3, *NS*) or a significant group x recording time interaction (F_1,20_  = 0.2, *NS*). In contrast, for mean wake bout durations, ANOVA revealed a significant main effect of recoding time (F_1,20_  = 4.8, P<.05), but not of group (F_1,20_  = 3.7, *NS*), and also no significant group x recording time interaction (F_1,20_  = 2.3, *NS*). Again, percentage of time awake at night was significantly greater in sham but not lesioned pups. ANOVA revealed no main effect of group (F_1,20_  = 0.1, *NS*) or recording time (F_1,20_  = 4.1, *NS*), and no significant interaction (F_1,20_  = 1.5, *NS*).

Figure 7B shows the extent of the SCN lesions. The smallest (green filled) and largest (yellow filled) for each section are shown. Adjacent areas were also damaged by the lesions in some pups, including parts of the optic chiasm and MPOA. Because the optic chiasm was damaged in some subjects, it is likely that some of the SCN-lesioned subjects were at least partially blind. However, we are confident that these lesions did not result in free-running rhythms because even bilateral enucleation at P3 (with recording through P35) or P11 (with recording through P21) did not impair the expression of day-night differences in sleep-wake cyclicity [3].

Four additional pups from 4 litters received electrolytic lesions that spared all cells within the SCN. In these cases, lesions were found in the MPOA and PVN. These “misses” exhibited day-night differences in sleep and wakefulness and power-law distributions of wakefulness that were very similar to those of shams.

A total of 8 SCN-lesioned pups died before recording at P21 (mortality rate  = 22.2%). Finally, no immediately obvious behavioral differences were detected in lesioned pups as compared to controls.

## Discussion

The present findings document how the SCN, DMH, and LC contribute to the development of the circadian control of arousal and the consolidation of wakefulness. We have shown that (i) SCN responsiveness to evoked arousing stimulation develops between P2 and P8 and is mediated by the LC; (ii) the emergence of LC-mediated activation of the SCN occurs contemporaneously with the development of bidirectional projections among the SCN, DMH, and LC; (iii) the SCN of P2 rats is critical for the day-night differences in sleep and wakefulness reported previously [3]; and (iv) precollicular transections at P2 do not eliminate these day-night differences, suggesting that the SCN or related structures (e.g., DMH) modulate sleep-wake behavior at this age via the release of humoral factors. We have also shown that (v) precollicular transections at P8 eliminate the day-night differences in sleep behavior normally observed at that age; and (vi) DMH lesions at P8 eliminate the day-night differences in wake behavior normally observed at P21. Finally, (vii) SCN lesions at P8 produced effects at P21 that were indistinguishable from those of DMH lesions, as would be expected if the SCN and DMH work closely together to modulate circadian sleep-wake behavior [37].

Using adult rats, Deboer et al. [8] showed that both active sleep and wakefulness are associated with *spontaneous* increases in SCN neural activity. In contrast, here we assessed whether arousing stimulation can *evoke* changes in SCN activity. Because Todd et al. [9] found, using P2 rats, that 30 min of arousing stimulation increased Fos-ir in the LC and DMH but not the SCN, we predicted that this same procedure would activate the SCN (in addition to the LC and DMH) later in development. Using P8 rats, this prediction was confirmed. In addition, destruction of LC noradrenergic terminals with DSP-4 [12]–[14] prevented evoked increases in SCN and DMH Fos-ir, thus indicating mediation by the LC. It is important to emphasize that DSP-4 is selective in its destruction of noradrenergic terminals arising from the LC [12]; destruction of these terminals likely leads to retrograde degeneration [38], resulting in the decreased Fos-ir in the LC observed here.

Given that SCN activity in adults increases spontaneously during both active sleep and wakefulness [8], it is interesting that the LC is nearly silent during active sleep but highly active during wakefulness in infant [39] and adult [40] rats. Accordingly, it may be that increased SCN activity during active sleep is mediated by an LC-independent pathway. In contrast, an LC-dependent pathway may activate the SCN during wakefulness; this LC-dependent pathway may be the same one that drives SCN activity in response to arousing stimulation in infants.

Although Deboer and colleagues [8], using adult rats, showed that spontaneous SCN activity increases during the transition from NREM to wakefulness, several studies report decreases in SCN activity during wake-related movements or after forced arousal (e.g., sleep deprivation) [41], [44], [45] (but see [42], [43] for differing results). Methodological differences most likely account for these differences. Regardless, the increase we observed in SCN Fos-ir at P8 was reliable and robust.

Similar variability in SCN responses to stress have been reported in adults [46], [47]. We addressed the issue of stress here by measuring Fos-ir in the PVN. Even during the stress-hyporesponsive period, which spans from P4 to P14 in rats and is characterized by a blunted adrenocortical response to stress, Fos-ir in the PVN can increase in response to a mild stressor such as saline injection [27]. However, as in our previous study using P2 rats [9] we found no evidence of PVN activation in either of the stimulated groups, thus suggesting that stress does not account for our results.

It is possible that it was the somatosensory aspect of the cold stimulation, rather than its arousing properties, that drove Fos-ir within the SCN at P8. Our results suggest otherwise. Specifically, whereas both barrel cortex and SCN were activated by cold stimulation to the snout, only SCN activity was blocked by DSP-4 (see Table 1). In light of the well-established role of the LC in behavioral arousal in adult rats [48]–[52], we conclude that barrel cortex responded to the somatosensory aspects of the cold stimulus and that the SCN responded to its arousing aspects.

As illustrated in Figure 8, the stimulation transmitted via the LC to the DMH and SCN appears to depend upon connections that develop between P2 and P8. We found sparse projections between the SCN and other downstream structures at P2. At P8, however, we found bidirectional projections among all three areas. This finding is consistent with work in hamsters showing that increased connectivity between the SCN and downstream structures first develops at P1 and continues through the end of the first postnatal week [53].

### Humoral factors

Despite sparse connectivity among the SCN, DMH, and LC at P2, infant rats exhibit day-night differences in sleep and wake bout durations [3]. To determine whether these effects depend upon the SCN, we lesioned the SCN at P1 and recorded pups for sleep and wakefulness during the day or night at P2. In contrast with shams, pups with SCN lesions no longer expressed day-night differences in sleep and wakefulness. Moreover, day-night differences in sleep and wakefulness were retained in P2 rats with precollicular transections that isolate the SCN from the brainstem. Therefore, the expression of circadian rhythmicity at P2 is attributable to the presence of a functioning SCN, perhaps exerting its behavioral effects via humoral factors at this age (see Figure 8).

Humoral factors released by the SCN modulate behavioral and physiological rhythms [54]. Several humoral factors have been identified in infants and adults, including transforming growth factor alpha (TGF-alpha) and prokineticin 2 (PK2) [55]–[61]. Both TGF-alpha and PK2 affect locomotor activity rhythms in adult mammals [56], [62] and, although not tested here, may be involved in modulating day-night differences in sleep and wakefulness at P2. To our knowledge, the present findings are the first to provide evidence, although indirect, of humoral modulation of sleep-wake rhythms in the early postnatal period.

Our findings also suggest that any humoral modulation of sleep-wake behavior that exists at P2 declines in efficacy over the first postnatal week. Specifically, we saw no evidence of humoral modulation of behavior in P8 rats with precollicular transections (that isolate the SCN and DMH from the brainstem) or P21 rats with DMH or SCN lesions. All together, these results strongly suggest a diminishing role for humoral factors in the modulation of sleep-wake behavior beyond the first postnatal week.

There are at least two hypotheses that could explain how humoral factors might lose their efficacy across early development. First, the developing direct neural connectivity described here may induce organizational changes in the SCN such that, by P8, the release of particular humoral factors is diminished. The fact that encapsulated SCN grafts can function humorally for months after implantation in the host [63] is not inconsistent with this hypothesis as encapsulation would prevent innvervation-related inductive interactions that could modify SCN function. Second, the development of neural connections in this system may mask any humoral influences on behavior that could remain in older animals.

With regard to the first hypothesis, it is interesting that, to our knowledge, only fetal and P1 SCN tissue has been effective in restoring behavioral circadian rhythms in SCN-lesioned adult hamsters [64], [65]. Moreover, the age of the donor influences the ability of SCN grafts to restore circadian rhythmicity in hamsters [65]: Whereas unencapsulated SCN grafts from embryonic or P1 subjects are equally effective in restoring circadian locomotor rhythms, grafts from older donors (P5-P10) are ineffective. On the other hand, the SCNs of P10–14 mice release paracrine signals *in vitro* that are sufficient to restore cellular synchrony and amplitude of pacemaking in SCN circuits lacking vasoactive intestinal peptide (VIP) [66]. Clearly, more work is needed to determine if there is an age-related decline in the SCN's capacity to release humoral factors and, if so, the mechanisms that mediate that decline.

### Wake bout consolidation and power-law distribution

We have shown previously that the highly fragmented bouts of sleep and wakefulness at P2 follow an exponential distribution and that, by P15, only wake bouts transition to a power-law distribution [3], [4]. This transition to a power-law wake distribution appears to require a functioning LC because treatment of infant rats with DSP-4 selectively prevents the expression of power-law behavior without producing fragmented bouts [36]. In contrast, as shown here, lesions of the DMH or SCN eliminated power-law wake behavior and also produced highly fragmented wake bouts. Sleep bouts were unaffected.

One possible interpretation of the above results is that consolidated wake bouts are necessary but not sufficient for the expression of power-law wake bout distributions. In other words, the loss of power-law wake distributions in pups with SCN and DMH lesions may have been a consequence of extreme fragmentation. Thus, there may be two discriminable forms of wake consolidation: One produced by a circuit that proceeds from the SCN to the DMH and into the brainstem–bypassing the LC–to produce consolidated wake bouts that follow an exponential distribution, and the other produced by a circuit that includes the LC and contributes to the expression of power-law wake bout distributions.

Surprisingly, of the many SCN lesion studies in mammals [67]–[69], only one has examined effects on sleep and wake bout durations. Using adult squirrel monkeys, Edgar et al. [70] showed that SCN lesions produced fragmented wake bouts without affecting sleep bouts. Similarly, in the present study, SCN lesions performed at P8, with recording at P21, produced fragmented wake bouts without affecting sleep bouts. These findings suggest that sleep bout consolidation is controlled by a separate forebrain circuit, perhaps including the ventrolateral preoptic nucleus and basal forebrain [31], [71]–[73]. However, it remains possible that the SCN contributes to sleep bout consolidation in rats at later ages when day-night differences in sleep are observed [3], [74].

## Conclusions

All together, the present results elucidate the neural mechanisms underlying the consolidation and circadian regulation of arousal in developing animals. Our results suggest a transition in the SCN's modulation of arousal–from humoral to non-humoral–corresponding with emerging bidirectional connectivity with downstream structures by P8. By P21, the SCN, DMH, and LC work together to make possible the extended periods of wakefulness necessary for the emerging independence that is a defining feature of weaning [75].
